# Roles of ethylene, jasmonic acid, and salicylic acid and their interactions in frankincense resin production in *Boswellia sacra* Flueck. trees

**DOI:** 10.1038/s41598-020-73993-2

**Published:** 2020-10-07

**Authors:** Fukuju Yamamoto, Fumiko Iwanaga, Ahmed Al-Busaidi, Norikazu Yamanaka

**Affiliations:** 1 Arid Land Research Center, Tottori University, Hamasaka, Tottori 1390 Japan; 2 Faculty of Agriculture, Tottori University, Minami 4-101, Koyama, Tottori Japan; 3 College of Agricultural and Marine Sciences, Sultan Qaboos University, Al Khoudh, Muscat 123 Oman

**Keywords:** Ecology, Plant sciences

## Abstract

The roles of ethylene, jasmonic acid, and salicylic acid and their interactions in frankincense resin production in *Boswellia sacra* trees growing in the drylands of Oman were studied. On March 18 (Experiment 1) and September 17 (Experiment 2), 2018, 32-year-old *B. sacra* trees with multiple trunks were selected at the Agricultural Experiment Station, Sultan Qaboos University, Oman. Various lanolin pastes containing Ethrel, an ethylene-releasing compound; methyl jasmonate; sodium salicylate; and combinations of these compounds were applied to debarked wounds 15 mm in diameter on the trunks. After a certain period, the frankincense resin secreted from each wound was harvested and weighed. The anatomical characteristics of the resin ducts were also studied in the bark tissue near the upper end of each wound. The combination of Ethrel and methyl jasmonate greatly enhanced frankincense resin production within 7 days in both seasons. The application of methyl jasmonate alone, sodium salicylate alone or a combination of both did not affect resin production. These findings suggest a high possibility of artificial enhancement of frankincense resin production by the combined application of Ethrel and methyl jasmonate to *B. sacra* trees.

## Introduction

*Boswellia sacra* Flueck., a dry-season-deciduous broadleaf tree with a height of 6 m, is distributed in Yemen, Oman, the southern Arabian Peninsula, Somalia and the Horn of Africa^1^. *B. sacra* is classified as “Near Threatened” on the IUCN Red List^2^ because of its poor regeneration in Oman. The major product of *Boswellia* species, a dried oleo-gum resin exuded from wounded stem surfaces, has been used as frankincense since ancient times^3,4^. In Dhofar Governorate, the main region of frankincense production in Oman, *B. sacra* trees are the most economically important plants, as the commercial and phytopharmaceutical source of frankincense production^5^.

In the Dhofar region, frankincense is harvested by tapping the trunks and branches of *B. sacra* trees, as for *B. papyrifera* in Ethiopia^4^. However, under current conditions, this tapping is frequently too aggressive, often leading to irreversible degradation of the tree^6^. To enable sustainable frankincense farming in Dhofar, optimizing the size, intensity and timing of tapping treatments for a given tree size is needed^6^. In addition, it is essential to establish efficient frankincense production techniques to achieve stable yields, rapid damage repair, and reduction of aftereffects on the entire tree. In view of the above factors, the roles of phytohormones, which are involved in the physiological mechanisms of plant defense responses^7^, should be elucidated, and a technology for the artificial control of resin secretion should be developed by using these signaling molecules.

Resin secretion in a damaged stem area caused by biotic or abiotic stimuli is called resinosis^8^. In contrast, an excessive outflow of gum such as gum Arabic production in *Acacia senegal*^9^ as a result of external stimuli is known as gummosis^10^. Such resin and gum secretions can isolate and seal off the damaged stem tissue, inhibiting desiccation and expansion of the injured area and preventing the secondary attack and spread of pathogenic microorganisms^11^. Frankincense production on the wounded bark of *Boswellia* species is a typical form of resinosis: a defense reaction facilitated by internal secretory structures called resin ducts, i.e., resin canals in the bark^4^. In the process of resin duct formation, three developmental types have been recognized: schizogenous, lysigenous, and schizolysigenous, which is characterized by traits of both the previous types^10^. Schizogenous ducts form through the separation of cells, resulting in a space lined with secretory cells composing an epithelium surrounding each resin duct. Lysigenous ducts result from the dissolution of cells^10^. In many angiosperms, vertical, tangential, and radial ducts form a continuous three-dimensional system connecting the leaves, stems, and roots throughout the plant^12^. Normal vertical and horizontal schizogenous resin ducts in angiosperms, e.g., Burseraceae, including *Boswellia* species, occur in the phloem^12^.

There are two types of resin ducts in woody plants: constitutive (or normal) resin ducts and inducible resin ducts known as traumatic resin ducts^10^. Constitutive resin ducts are commonly observed in the phloem and/or xylem of coniferous species and function in structural defense^10,13–16^. Such constitutive resin ducts, similar to those of conifers, have also been observed in many families of woody angiosperms, such as Anacardiaceae, Asteraceae, Brassicaceae, Fabaceae, Hypericaceae, Simaroubaceae^10^ and Burseraceae, including *Boswellia* species^4,12^.

The development of traumatic resin ducts as an inducible defense response occurs in the phloem and/or xylem tissues of many coniferous species^12,17,18^ and mostly in the phloem of woody angiosperm species^4,12^ after challenge by external stimuli, including mechanical wounding, insect attacks and infection by microorganisms. In most plants, the diverse defense mechanisms occurring in wounded tissue in response to various external stimuli are regulated through a complex network of signaling pathways mediated by plant hormones. Among these plant hormones, ethylene, jasmonate and salicylic acid (SA) are well recognized as elicitors, controlling the signaling pathways involved in the defense reactions of damaged plant organs, including resinosis and gummosis, that allow plants to repel secondary attack by insects and pathogens^7,19,20^. For instance, the induction of ethylene biosynthesis is likely correlated with the formation of traumatic resin ducts, which are not constitutive but inducible secretory structures^10^, in response to injury and pathogenic infection in the stems of various conifers^21^. Methyl jasmonate (MJ) has also been proposed to induce physical and chemical defenses related to wounding and fungal infection in the stems of several coniferous species, including the formation of traumatic resin ducts^16,18,22^. SA, a key signaling component that is required for locally and systemically induced resistance responses in plants following infection by pathogens^23,24^, plays an important role in many plant-pathogen interactions by activating defense responses^25^.

In many pathological studies of crop plants, researchers have shown that these three substances are the major plant hormones modulating plant defense responses^24,26^. For instance, ethylene and MJ synergistically regulate defense genes in many plants, such as tomato, tobacco, and *Arabidopsis*^27–29^. In *Arabidopsis*, jasmonic acid and ethylene activate plant genes involved in resistance to necrotic pathogens and wounding/mechanical stimuli^30^, whereas SA, another defense-related signaling agent, enhances resistance to biotrophic pathogens and promotes the expression of genes encoding pathogenesis-related proteins in host plants^23,31^. Ethylene, jasmonates and SA and their crosstalk are likely to be involved in a complex signaling network in which the different pathways influence each other through synergistic or antagonistic regulatory interactions^20,23,24,32^.

Various investigations have attempted to reveal synergistic or antagonistic regulatory interactions among these three substances by applying them externally. In coniferous species, application of ethylene or MJ alone, mimicking wound/infection stimuli, induces anatomically based defense responses such as the formation of traumatic resin ducts in the xylem of *Pinus taeda*^33^ and *Pn. densiflora*^34^ and in the secondary phloem of the Cupressaceae family^35^. Furthermore, a strong correlation between ethylene and MJ has been shown by Hudgins and Franceschi^18^, who found that MJ was efficient in inducing ethylene evolution. Treatment with an ethylene inhibitor, 1-methylcyclopropene (1-MCP), decreased MJ or the induction by wounding of traumatic resin duct development in the stems of *Pseudotsuga menziesii*, suggesting that MJ-induced responses were likely mediated by ethylene as a downstream signaling agent.

On the other hand, negative and positive interactions of SA with ethylene- or jasmonate-induced defense responses have been reported in a wide variety of crop plants^28,29,36^. However, plant defense mechanisms are highly complex, and these three plant hormones often show minimal or variable effects on the same pathway in different species.

There are several reports revealing the relationship between SA and jasmonic acid in the formation of traumatic resin ducts in conifer species. Kozlowski et al. found that SA levels increase when *Picea abies* is exposed to MJ, indicating a significant role of MJ in induced SA accumulation^37^. Hudgins and Franceschi reported that exogenous MJ and ethylene but not methyl salicylate caused enhanced phenolic synthesis in polyphenolic parenchyma cells, early sclereid lignification, and reprogramming of the cambial zone to form traumatic resin ducts in *Ps. menziesii* and *Sequoiadendron giganteum*^18^*.* Furthermore, jasmonic acid and SA were implicated in local and systemic responses of *Pn. banksiana* and *Pn. contorta* to *Grosmannia clavigera*, a sac fungus causing blue staining in wood, with SA appearing to play a greater role in response to *G. clavigera* in *Pn. banksiana* than *Pn. contorta*^38^. However, after reviewing the above reports, whether an antagonistic or synergistic relationship between SA and jasmonic acid can be observed in the stems of injured or microorganism-infected woody species remains unclear.

According to a report by Khan et al., the appropriate number of tapping points for frankincense resin production in *B. sacra* trees depends on trunk diameter, but the resin yield per tree per season depends on the size and age of the tree and the time of year^5^. If the demand for frankincense increases, tapping point number and frequency may be increased in individual trees, impacting tree growth and physiology. Furthermore, the intense stimulation resulting from tapping injuries may affect the overall production capacity of the tree. Khan et al. analyzed changes in various phytohormones, including gibberellic acid, indole-acetic acid, SA and kinetin, in leaves to study the responses of trees to incisions, which activate defense mechanisms through the systemic production of phytohormones to reduce the negative impacts of resin production^5^. However, the role of local phytohormonal changes on the physiological functions associated with frankincense secretion at the tapping site has not yet been analyzed.

Based on the investigations above, ethylene, MJ, and SA and their interactions are likely to be important in frankincense resin production at stem wounds in *Boswellia* species. However, there is little information about the physiological mechanism of frankincense production in relation to the roles of these hormones and their crosstalk.

The objectives of this study were to examine the effects of external application of Ethrel (Et), an ethylene releasing compound; MJ, sodium salicylate (NS); and combinations of these three compounds to stem wounds on frankincense production in *B. sacra* trees. Furthermore, this study aimed to elucidate the mechanisms of frankincense secretion and other injury responses in tree trunks, as well as to contribute to the establishment of technology for the artificial control of frankincense production.

## Results

### Observation of resin secretion after wounding and hormone treatment

**Figure 1:**
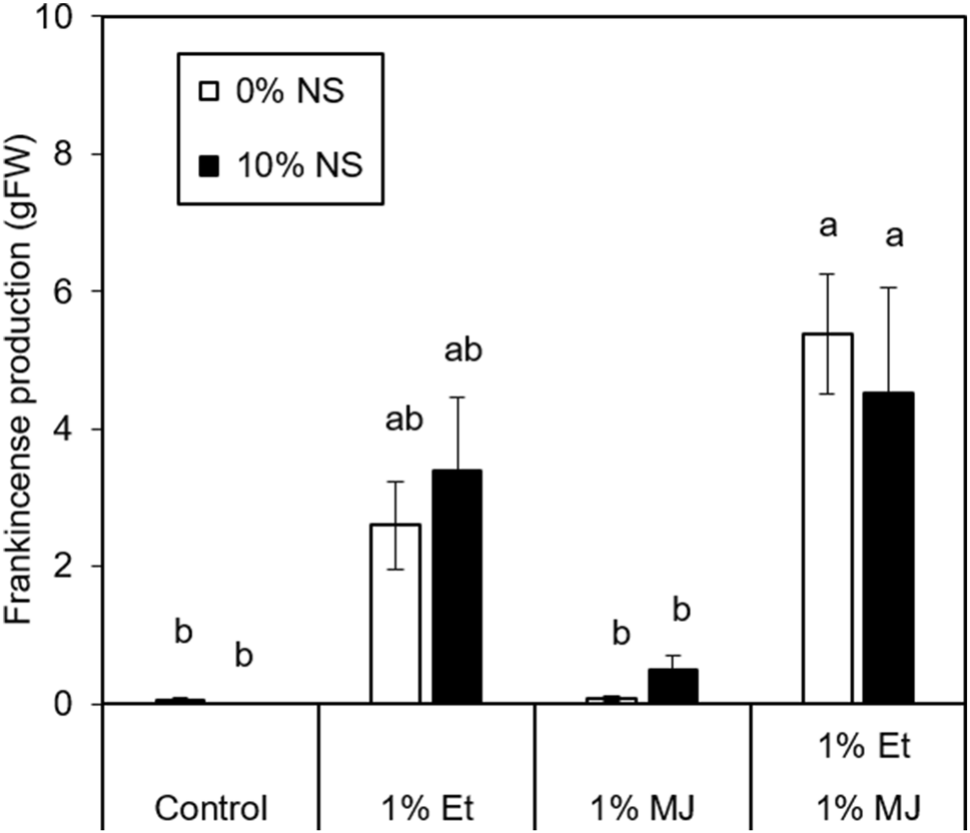
Effects of applied Ethrel (Et), methyl jasmonate (MJ), sodium salicylate (NS) and their combinations in dehydrated lanolin on frankincense resin exudation in *B. sacra* trees on the 23rd day after paste application in Experiment 1. Letters indicate significant differences according to Tukey’s HSD test (*p* < 0.05).

The application of 1% Et + 1% MJ and 1% Et + 1% MJ + 10% NS significantly increased frankincense resin exudation in March (Experiment 1, Fig. 1). In contrast, the application of 1% MJ alone, 10% NS alone or 1% MJ + 10% NS did not affect resin exudation.

**Figure 2:**
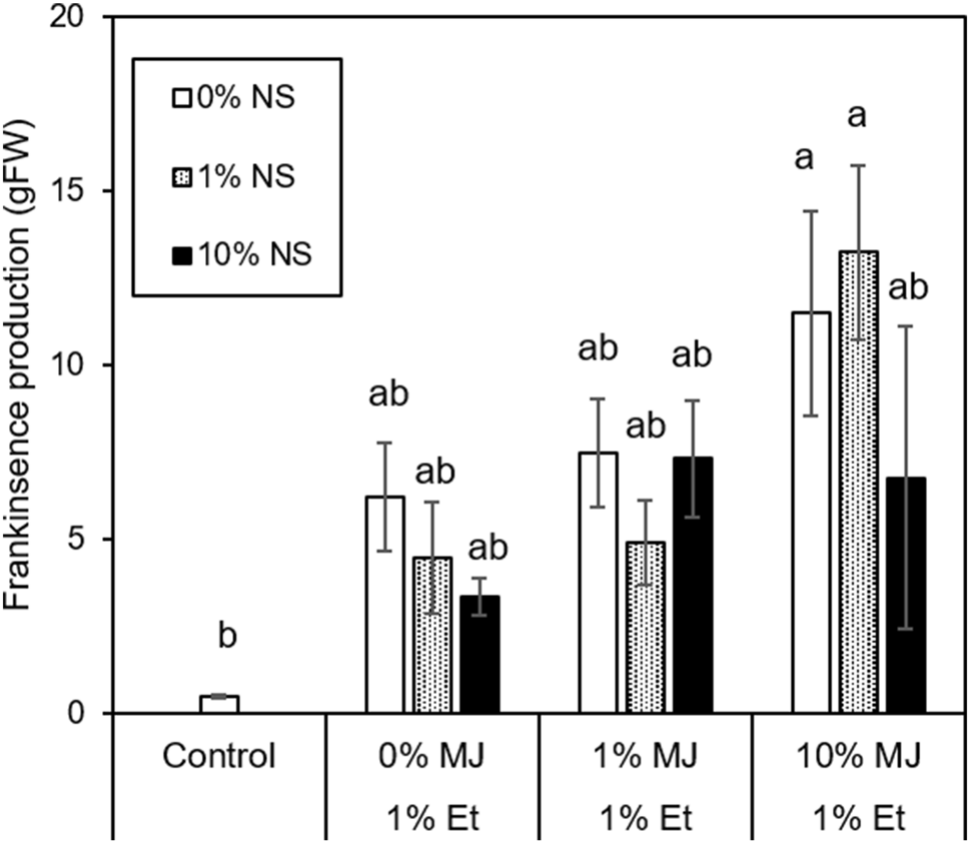
Effects of applied Et, MJ, NS and their combinations in dehydrated lanolin on frankincense resin exudation in *B. sacra* trees on the 6th day after paste application in Experiment 2. Letters indicate significant differences according to Tukey’s HSD test (*p* < 0.05).

**Figure 3:**
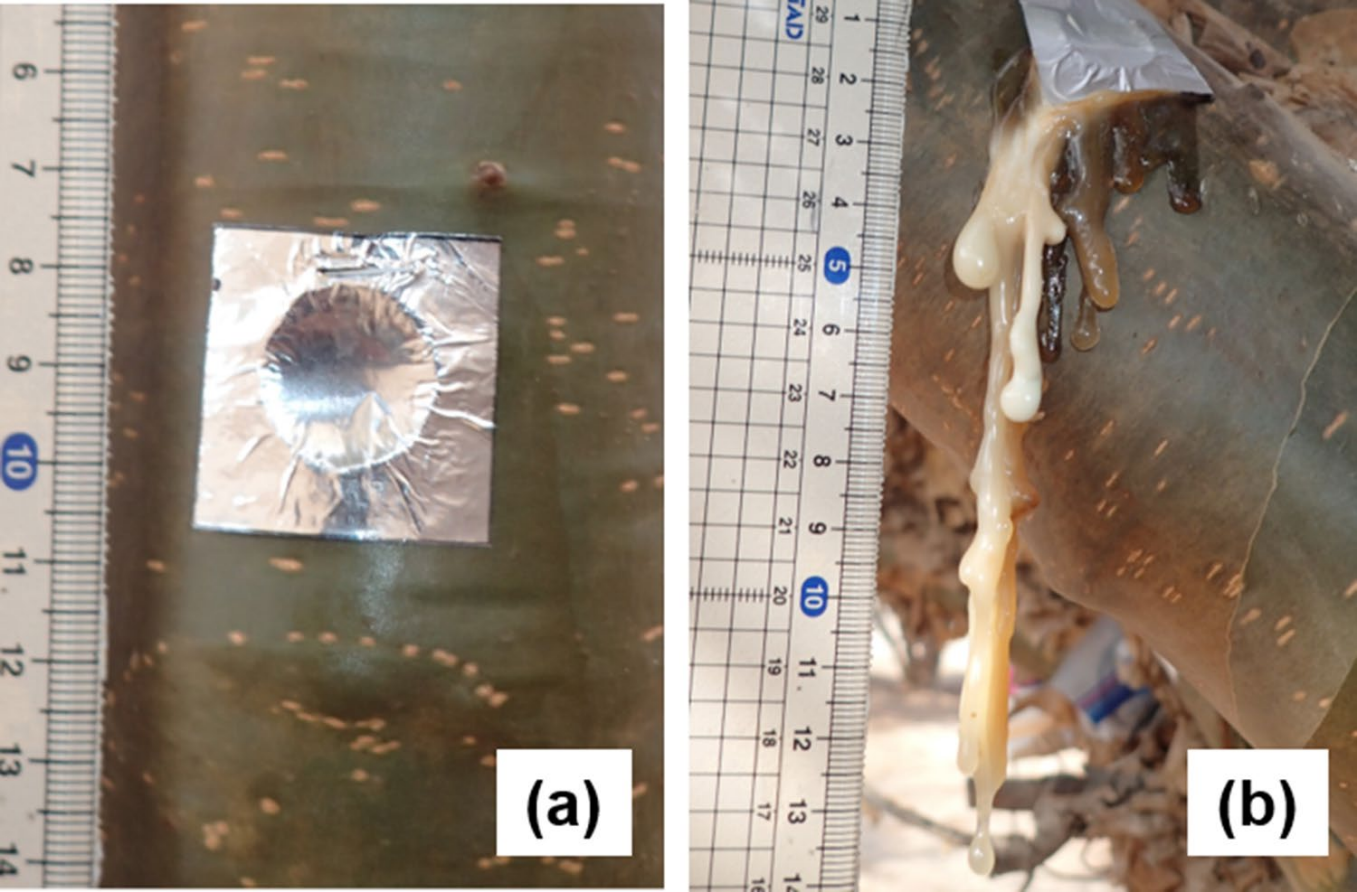
Frankincense resin exudation from debarked wounds on the control (left) and the 1% Et + 10% MJ + 1% NS (right) treatment within 144 h in Experiment 2.

In September (Experiment 2), combined application of 1% Et + 10% MJ and 1% Et + 10% MJ + 1% NS significantly enhanced resin exudation (Figs. 2, 3). Other treatments, such as 1% Et, 1% Et + 1% MJ, 1% Et + 1% NS, 1% Et + 1% MJ + 1% NS, 1% E + 10% NS, 1% Et + 1% MJ + 10% NS and 1% Et + 10% MJ + 10% NS, tended to promote resin exudation in comparison with the control, although the results were not significant. In Experiment 1, single treatments with Et or MJ and a combined treatment with Et + MJ were carried out. The results showed that both treatment with Et alone and combined treatment with Et + MJ tended to promote increased resin production (Fig. 1). Furthermore, when 10% NS was added to Et or MJ, there was no significant difference in resin production (Fig. 1).

In the September experiment (Experiment 2, Fig. 2), the effects of different concentrations of SA and MJ on resin duct formation were examined. In the case of the combined Et and SA treatment, there was no clear difference between increased SA concentration and resin duct formation. The inhibitory effect was also not clear when SA was added to the Et + 1% MJ treatment. Only when 10% NS was added to the Et + 10% MJ treatment did resin duct formation tend to be suppressed. Overall, the inhibitory effect of NS treatment on the effects of Et or MJ treatment was not significant.

Comparing the results of Experiment 1 in March with those of Experiment 2 in September, no significant differences were observed. For example, when comparing the combined Et and MJ treatment with Et alone, a tendency toward increased resin duct area at 1% ET + 1% MJ compared to Et alone was observed in both Experiments 1 and 2, but the difference was not statistically significant (Figs. 1,2). Based on these findings, no significant seasonal difference in the effect of plant hormone treatment at low concentrations on resin duct formation is expected.

**Figure 4:**
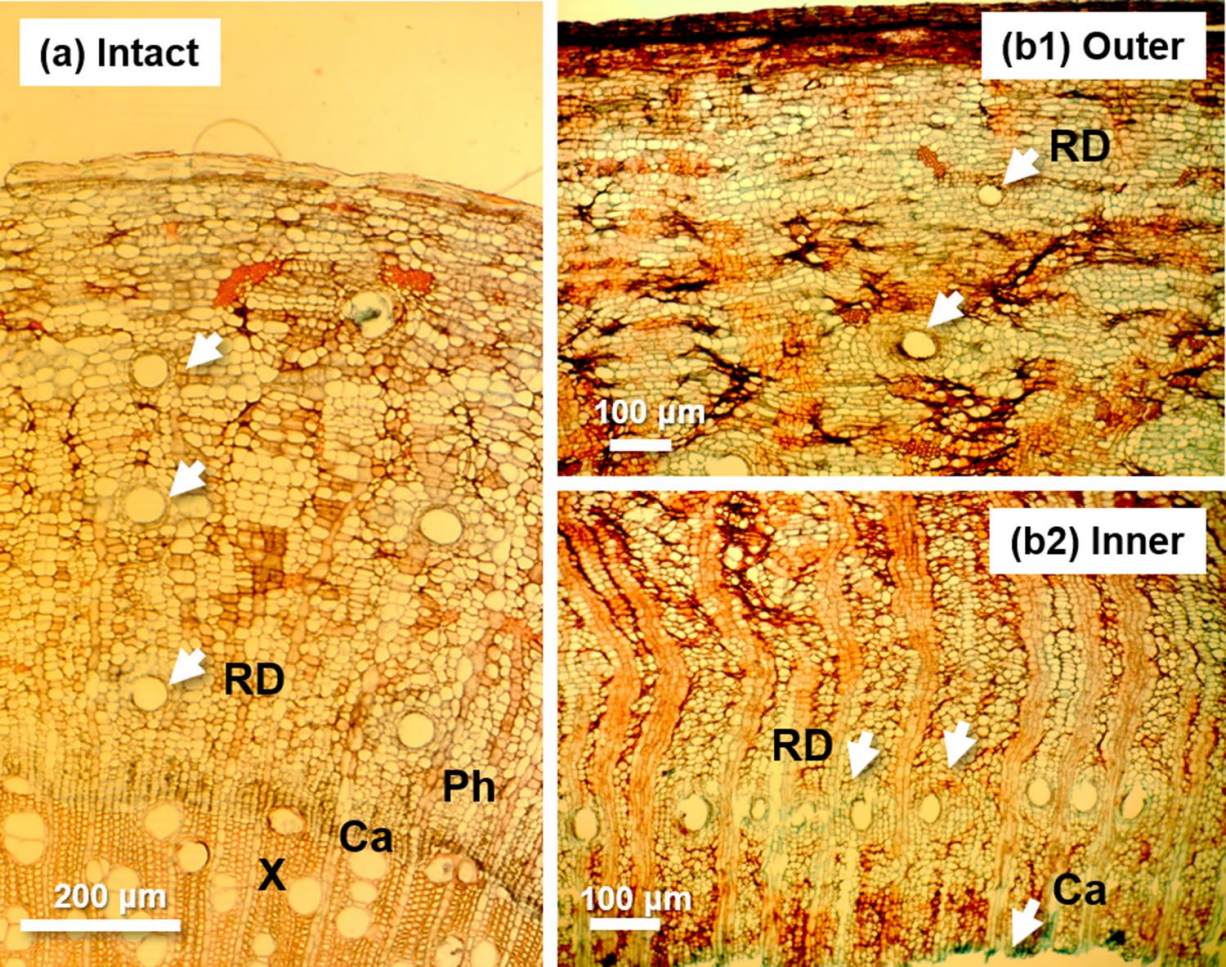
Transverse section of the bark of intact (**a**) and treated with 1% Et + 10% MJ + 1% NS (outer, **b1**; inner, **b2**) *B. sacra* trees in Experiment 2. Arrows showing resin ducts (RD) in the bark area. *Ca* cambial region, *Ph* phloem, *X* xylem.

Figure 4a indicates the transverse structure of intact bark in a *B. sacra* tree, showing scattered normal resin ducts, in Experiment 2. Wounding stress greatly affected the density and distribution of resin ducts in the bark. Figure 4b1,2 show the outer and inner bark tissues at 5 mm above the edges of debarked wounds treated with lanolin paste containing 1% Et + 10% MJ + 1% NS, respectively. The resin ducts in the outer part of the bark were low in density and dispersed (Fig. 4b1), whereas those in the inner part of the bark (periderm), which contains conducting phloem, occurred in a tangential series (Fig. 4b2).

### Anatomical changes after wounding and hormone treatment

As shown in Table 1, the resin duct density in Experiment 2 was compared between the outer and inner bark tissues at 5 mm above the edges of the debarked wounds treated with plain lanolin, 1% Et, 1% Et + 1% MJ, 1% Et + 1% MJ + 1% NS, 1% Et + 10% MJ + 1% NS and 1% E + 10% MJ + 10% NS. The intact control data were taken from transverse sections of unwounded bark collected at the beginning of the experiment. There was no significant difference in resin duct density between the outer and inner tissues of the intact bark. However, the resin duct density of the inner bark tissues was significantly higher than that of the outer tissue in most of the bark samples in contact with paste-treated wounds, regardless of hormone content. Even in the bark samples near the plain lanolin-treated wounds (control), a clear increase in the resin duct density of the inner bark tissues was observed. There was no significant difference in the resin duct density of either the outer or inner bark tissues among the various hormone treatments.

**Table 1:** Resin duct density in the outer and inner bark tissue of transverse sections taken at 5 mm above the edge of the hormone-treated wound in Experiment 2.

	Outer	Inner	
Intact	0.48 ± 0.14	0.51 ± 0.10	NS
Control	0.44 ± 0.07	0.85 ± 0.12	*
1E	0.33 ± 0.07	0.84 ± 0.20	*
1E, 1 MJ	0.70 ± 0.13	0.84 ± 0.11	NS
1E, 1 MJ, 1NS	0.52 ± 0.10	0.85 ± 0.08	*
1E, 10 MJ, 1NS	0.46 ± 0.12	0.81 ± 0.10	*
1E, 10 MJ, 10NS	0.43 ± 0.08	1.02 ± 0.15	*

Value shows mean ± standard error (n = 7).

1% Et (1E), 1% Et + 1% MJ (1E, 1 MJ), 1% Et + 1% MJ + 1% NS (1E, 1 MJ, 1NS), 1% Et + 10% MJ + 1% NS (1E, 10 MJ, 1NS) and 1% Et + 10% MJ + 10% NS (1E, 10 MJ, 10NS).

*Significant at *p* < 0.05; *NS* not significant (t-test, n = 7).

## Discussion

In the stems of woody plants, bark is a functional exterior organ that protects against various biotic and abiotic hazards, including mechanical wounding and microbial infection^10^. Various structural and chemical defense components in bark mitigate damage, while the rhytidome and periderm function as protective barriers to interfere with penetration and degradation. The chemical defenses include substances with toxic or inhibitory effects such as defensive proteins, enzymes and exudates such as resin, gum, kino and latex^19^.

Frankincense resin production on the wounded bark tissue of *Boswellia* species is a typical resinosis, a defensive manifestation induced by mechanical injury stimuli^4^. Constitutive and inducible defense mechanisms in stems to protect against injury are well demonstrated in the phloem and xylem cells of conifers^39,40^. These constitutive reactions include the secretion of resin from preformed resin reservoirs in *Pc. abies*^14,16^. Constitutive resin ducts similar to those of conifers occur in angiosperm families such as Anacardiaceae, Asteraceae, Brassicaceae, Fabaceae, Hypericaceae and Simaroubaceae^10^. *Boswellia* species, in the Burseraceae family, also have well-developed constitutive resin ducts. In *B. papyrifera* trees, frankincense resin is produced through resin ducts, which form a three-dimensional network within the inner bark^4^. In the present study, an increase in resin duct formation in a tangential series in the inner bark tissue of *B. sacra* trees was observed in every bark sample near a wound. Notably, traumatic resin ducts occur in tangential series in various coniferous species^10,13^. The present results suggest that the increased resin ducts in this study are traumatic resin ducts, not normal or constitutive resin ducts, as observed in the outer bark tissue. In many angiosperms, the vertical, tangential, and radial ducts in the phloem form a continuous three-dimensional system connecting the leaves, stems, and roots throughout the plant^12^.

According to the World Weather Information Service for Salalah, Oman^41^, the rainy season in the Dhofar region, where frankincense production flourishes, is affected by the monsoon from mid-June to mid-September. Meanwhile, the harvest of frankincense from *B. sacra* trees in Dhofar Governorate takes place during the dry season, between November and May^6^. In Ethiopia, frankincense production by *B. papyrifera* also takes place during the dry season, which lasts for approximately 8 months^4^. Because frankincense is produced by scarring the trunk of the tree, active growth during the rainy season is important for the trees to recover from wounds and overall damage. In this study, there was no significant difference in experimental results between March, during the frankincense-producing season, and September, during the nonproducing season. However, because the trees used in these experiments were maintained by irrigation, the conditions were not the same as those of the trees in Dhofar, which have different growing conditions during the dry and wet seasons. To rigorously examine the seasonality of tree trunk injury, it would be necessary to conduct experiments in Dhofar. However, there appears to be little seasonal variation in the interactive effects of ethylene and MJ treatments on the production of frankincense resin.

The diverse defense mechanisms of plants in response to various external stimuli are regulated through a complex network of signaling pathways. Ethylene, jasmonates and salicylates play important roles in regulating developmental processes and the signaling networks involved in plant responses to a wide range of external stimuli^7^. Ethylene regulates many important functions in plants, including cell differentiation, growth, development, senescence and response to various disturbances or stresses^42^. In the stems of various woody plants, ethylene is produced in response to various stimuli, including wounding, flooding^34,43^, gravity^44^, chemical administration^18^, and insect and pathogen attack^20,45,46^. Induction of ethylene biosynthesis is likely correlated with the formation of traumatic resin ducts, which is common in most conifer stems in response to injury and pathogenic infection^21^. Several reports have indicated that ethylene induces many traumatic resin ducts in the xylem of *Pn. taeda*^33^ and *Pn. densiflora*^47^ seedlings.

Jasmonates can activate defense genes, and wounding or elicitors can lead to the accumulation of jasmonates in plants^30^. Studies have revealed that applications of MJ increases the resistance of *Pc. abies*^37^ and *Pn. sylvestris*^48^ against biotic attacks. MJ has also been proposed to induce the same physical and chemical defenses as wounding and fungal infection, including the formation of traumatic resin ducts, in the stems of several coniferous species^16–18,22^.

SA is a key signaling component that is required for locally and systemically induced resistance responses in plants following infection by pathogens^23,24^. Endogenous synthesis or exogenous application of SA has been demonstrated to trigger pathogenesis-related protein transcription and resistance to a broad range of virulent pathogens in tobacco and Arabidopsis tissues^49–51^. In coniferous species, SA accumulation has been shown in the roots of *Pc. abies* trees after fungal inoculation^37,52^. Davis et al. subsequently found that chitinase activities, which are considered markers for induced defense reactions, were induced in *Pn. elliottii* after challenge by pathogens and SA^53^.

Ethylene, jasmonates and SA are involved in a complex signaling network in which the different pathways influence each other through synergistic or antagonistic regulatory interactions^20,23,24,32^. For instance, ethylene and MJ synergistically regulate defense genes in many plants, such as tomato, tobacco, and Arabidopsis^27–29^. In woody plants, Hudgins and Franceschi demonstrated that ethylene production increased in *Ps. menziesii* following the application of MJ, and application of an ethylene inhibitor could inhibit response to MJ^18^. Their findings indicate that MJ-induced responses are mediated by ethylene.

The induction of cell death in response to pathogen infection in tomato requires both ethylene and SA, and the accumulation of SA in infected tissues is dependent on ethylene biosynthesis^54^. In contrast, SA appears to inhibit jasmonic-acid-induced expression of defense genes in tomato^55^. In woody plants, Kozlowski et al. reported that MJ induced the accumulation of free SA in all parts of *Pc. abies* seedlings^37^. However, in other tree species, SA has been reported to be less involved in injury and disease response than ethylene and jasmonic acid. Hudgins and Franceschi have reported that exogenous MJ and ethylene but not methyl salicylate caused enhanced phenolic synthesis in polyphenolic parenchyma cells, early sclereid lignification, and reprogramming of the cambial zone to form traumatic resin ducts in *Ps. menziesii* and *S. giganteum* seedlings^18^. Although the roles of these three signaling molecules in defense against external stimuli seem to be essential, their synergistic or antagonistic interactions are complex and vary by species. In our present results, trunk wounds on *B. sacra* trees exhibited a rapid increase in frankincense resin production from the bark after the application of combined Et and MJ, suggesting an interaction between ethylene and jasmonic acid in the resinosis of this species. However, SA seems to be less important in *B. sacra* resinosis.

Resin duct formation occurred in every bark sample taken near debarked wounds, even in the samples near the control wounds. These results indicate that the roles of ethylene and jasmonate and their interactions in resin duct formation are still obscure. However, in practical terms, there is a high possibility of artificial enhancement of frankincense resin production by the combined application of Et and MJ to trunk wounds on *B. sacra* trees. More precise analysis, such as determining the optimal concentrations for the combination of these substances, will be needed to enhance frankincense resin production. Furthermore, the essential oil of frankincense contains mainly n-octyl acetate, octanol and limonene^3,56^. The quality of the frankincense resin promoted by the application of plant hormones in the present study is an important issue to be clarified in the future.

## Materials and methods

The harvest of frankincense from *Boswellia sacra* trees in Dhofar Governorate, Oman, takes place between November and May^6^. Therefore, considering the different physiological conditions of the trees, two experiments were conducted, one in March 2018, during the frankincense harvesting season (Experiment 1), and the other in September 2018, during the nonharvesting season (Experiment 2).

### Study site

The experiments examining the promotion of frankincense secretion were conducted in the garden of the Agricultural Experiment Station, Sultan Qaboos University, Muscat, Sultanate of Oman. The location of the experiments was 23°35′57″ N and 58°09′55″ E at an altitude of 50 m. The soil properties of the experimental site were as follows: EC, 2 dS/m; pH, 8.2; texture, sandy loam. Each tree was irrigated with 100–200 L of water once daily, and organic fertilizer was applied annually to maintain tree growth.

The averaged meteorological information for Muscat from 1981 to 2010 was obtained from the Global Historical Climatology Network (GHCN) data^57^ for the Muscat International Airport (23°35′30″ N and 58°16′45″ E) location: the mean daily, mean maximum, and mean minimum temperatures for March 2018 were 26.2 °C, 30.9 °C, and 21.3 °C, respectively. Those for September were 30.5 °C, 34.5 °C and 27.6 °C, respectively. No precipitation was observed in either March or September.

### Plant materials

**Figure 5:**
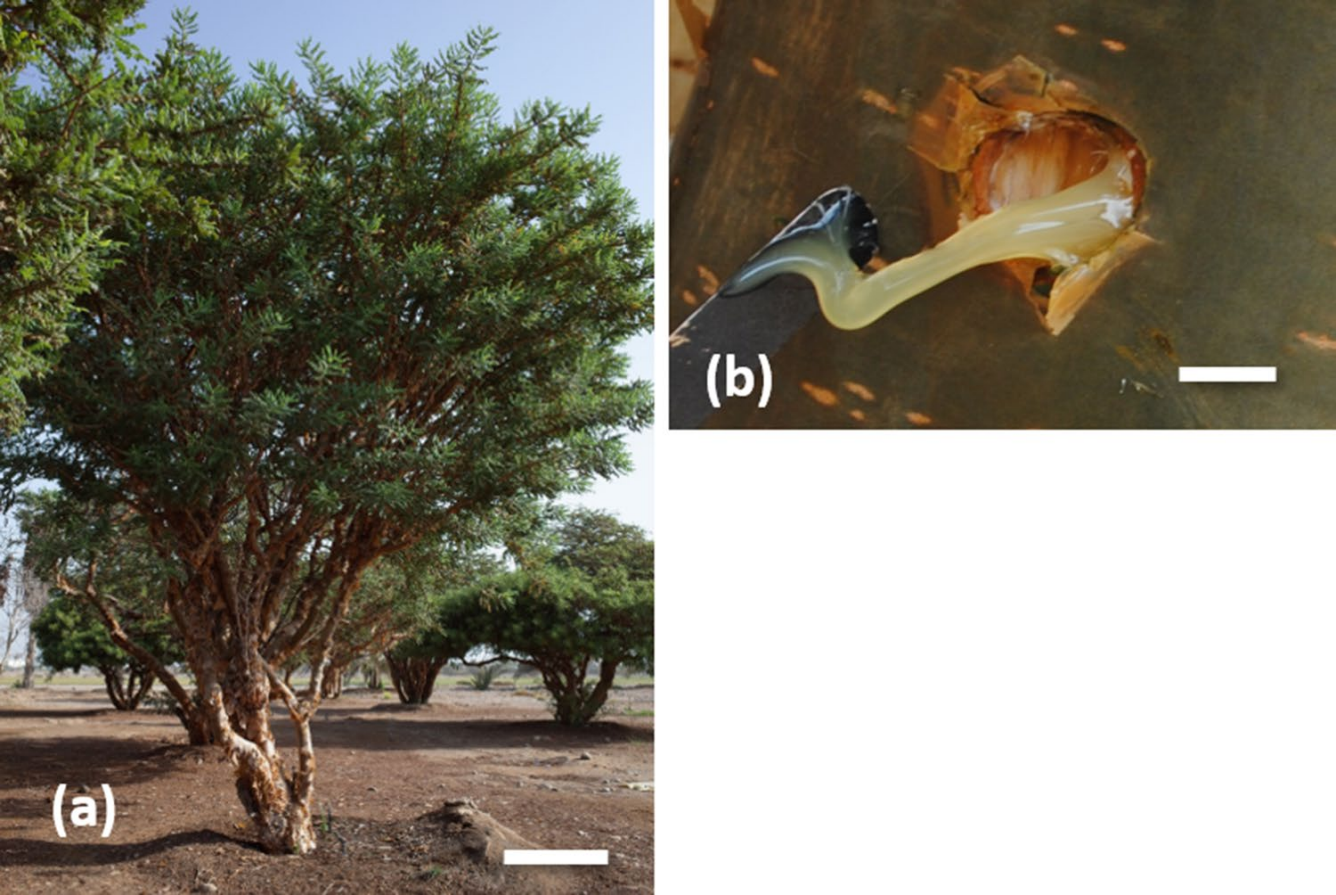
Condition of a 32-year-old *Boswellia sacra* tree with multiple trunks growing on the campus of Sultan Qaboos University, Muscat, Oman (**a**), and bark wounds treated with lanolin paste containing hormones (**b**). Both photos were taken on September 16, 2018, in Experiment 2. The vertical bar indicates 1 m (**a**) and 10 mm (**b**), respectively.

In the University frankincense garden, frankincense trees having multiple trunks without traces of tapping were selected for uniformity of size and development and used for either Experiment 1 or Experiment 2 (Fig. 5a). The average tree heights, heights at wounding locations and trunk diameters were 5.2 ± 0.4 m, 156.1 ± 2.6 cm and 10.1 ± 0.4 cm, respectively.

### Experiment 1

Six trees were used for Experiment 1. On March 18, 2018, eight trunks were selected from among the multiple trunks of each tree and used for 8 different paste treatments. The application of plant hormones mixed with lanolin allows them to penetrate at much lower concentrations into the internal tissues surrounding the wounds (Fig. 5b). For this reason, the concentrations were adjusted in percentage order according to the results of a previous study conducted with *Acacia seyal* for gummosis^58^. Eight different types of lanolin paste containing Et, MJ and NS were prepared just before treatments: (1) plain dehydrated lanolin as a control; (2) 1% Et; (3) 1% MJ; (4) 1% Et + 1% MJ; (5) 10% NS; (6) 1% Et + 10% NS; (7) 1% MJ + 10% NS; and (8) 1% Et + 1% MJ + 10% NS. The hormones were combined with dehydrated lanolin and several drops of Tween 20, a surfactant. Each concentration was expressed by weight ratio. A small circular bark segment was removed from the surface of each trunk with a leather punch (15 mm in diameter) and a hammer to produce a debarked wound. Approximately 0.5 g of lanolin paste was applied to the debarked wound on each trunk with a spatula (Fig. 4). Each paste-treated wound was covered with a piece of aluminum foil (3 cm × 3 cm) to protect it from sunlight and dehydration. On April 10th, when 23 days had passed after the initiation of the experiment, the exuded frankincense resin was harvested from each of the paste-treated wounds and weighed immediately.

### Experiment 2

On September 17, 2018, seven trees were selected in the same garden of the campus. Ten trunks among the multiple trunks of each tree were selected and used for 10 different paste treatments. The concentrations and combinations of plant hormones were slightly modified in Experiment 2, taking into account the results obtained in Experiment 1. Since the interaction between MJ and NS was not clear in Experiment 1, we increased the concentration of MJ. Furthermore, the concentration of MJ was also adjusted to examine its concentration dependence, because an interaction between Et and MJ was suggested by the results of Experiment 1. Ten different types of lanolin paste containing Et, MJ and NS were prepared just before the treatments: (1) plain dehydrated lanolin as a control; (2) 1% Et; (3) 1% Et + 1% MJ; (4) 1% Et + 10% MJ; (5) 1% Et + 1% NS; (6) 1% Et + 1% MJ + 1% NS; (7) 1% Et + 10% MJ + 1% NS; (8) 1% Et + 10% NS; (9) 1% Et + 1% MJ + 10% NS; (10) 1% Et + 10% MJ + 10% NS. The hormones were prepared and applied to debarked wounds as described above. On Sept. 23, when 6 days had passed after the initiation of the experiment, the exuded frankincense resin was harvested from each of the paste-treated wounds and weighed immediately.

After the resin was collected, one circular bark segment (15 mm in diameter) in contact with the upper end of each paste-treated wound was sampled for anatomical analysis with the same leather punch. Each bark sample was soaked in 70% ethanol solution for fixation and sterilization. After 24 h, the samples were removed from the ethanol solution, packed in plastic bags and transported to a laboratory at Tottori University, Japan.

The samples were sectioned transversely at 5 mm above the upper end of each wound with a thickness of 15 μm on a sliding microtome. The bark sections were stained with safranin-fast green solution and mounted in Diatex. For each section, the structure of the resin ducts and their density were studied with light microscopy. The density of the resin ducts was measured in the outer and inner layers of the bark of each section on digital photos taken with a rectangular area of 3 mm tangential and 2.2 mm radial. The resin duct area was measured on each digital photo with DP2-BSW software (Olympus Corp., Tokyo, Japan). To test for differences in resin production among the treatments, a statistical analysis of the experimental results was performed using Tukey’s HSD test. The statistical analysis was performed using R software (ver. 3.5.3).

## Data Availability

The datasets used in the current study are available from the corresponding author on reasonable request.
